# Congenital Insensitivity to Pain: A Case Study of a Rare Genetic Disorder

**DOI:** 10.7759/cureus.69414

**Published:** 2024-09-14

**Authors:** Nurhan N Al-Hroub, Ala'a A Al-Salahat, Manwa A Taamreh, Fawzy M Abunejma, Osama N Dukmak

**Affiliations:** 1 Medicine, Al-Quds University, Bethlehem, PSE; 2 Pediatrics, Faculty of Medicine, Hebron University, Hebron, PSE; 3 Pediatric Department, Palestinian Red Crescent Society Hospital, Pediatrics, Hebron, PSE; 4 Surgery, School of Medicine, Al-Quds University, Jerusalem, PSE

**Keywords:** alpha subunit 9, charcot joints, congenital insensitivity to pain, nonsense mutation, sodium channel nav1.7

## Abstract

Congenital insensitivity to pain (CIP) is an exceedingly rare autosomal recessive condition caused by *SCN9A* Nav1.7 loss-of-function mutations. We present a case of a patient with clinical symptoms compatible with CIP who had a homozygous *SCN9A* probable pathogenic variation, which results in a premature stop codon. According to the recommendations of the American College of Medical Genetics and Genomics, it is classified as probable pathogenic (class 2).

Early detection and treatment may aid in reducing mortality and morbidity as the signs and symptoms of CIP with dysmorphic features manifest early, typically at birth or during infancy. However, with careful medical attention, affected individuals can have longer life expectancies.

## Introduction

The *SCN9A* gene encodes instructions for the production of one component (the alpha subunit) of the NaV1.7 sodium channel [1]. Sodium channels transfer positively charged sodium atoms (sodium ions) into cells and serve an important role in the generation and transmission of electrical signals [1]. NaV1.7 sodium channels are located in nociceptors, which are nerve cells that carry pain signals to the spinal cord and brain [1]. In addition, olfactory sensory neurons, nerve cells in the nasal cavity that send signals to the brain related to smell, contain the NaV1.7 channel [1].

Congenital insensitivity to pain (CIP) is caused by mutations in the *SCN9A* gene, which produce nonfunctional alpha subunits that cannot be integrated into NaV1.7 channels, and, as a result, the channels cannot form.

Due to this lack of pain sensitivity, injuries, bruising, broken bones, and other health problems frequently build up and may go unnoticed. Young children with CIP with anhidrosis (CIPA) may repeatedly self-bite, leading to mouth or finger wounds as well as various burn-related injuries, which will lead to shorter life expectancies. Also, CIP is frequently accompanied by an absence of smell (anosmia) [2].

## Case presentation

A 13-year-old female adolescent with noticeable minor facial dysmorphic features (broad nasal bridge, the mouth appears large, and a thin upper lip) was investigated for a possible genetic disorder in light of her history, which goes back to 10 years at least when she developed recurrent episodes of febrile joints swelling (ankles and right knee), which lasted each time for almost two weeks approximately.

She had a history of insensitivity to pain during vaccination. When she was two years old and learning to walk, her parents noted that she had multiple falls, as well as left ankle edema, associated with recurrent fever (around 39°C). Also, the parents observed non-pus blisters on the tip of the finger, which were treated as burns.

Then she remained symptom-free for one year, up until the age of three when she began complaining of similar occurrences in her right ankle. At this point, she was diagnosed with osteomyelitis, which was treated, with only partial improvement. She still had persistent edema and periodic fever bouts and received nonsteroidal anti-inflammatory drug as required.

**Figure 1:**
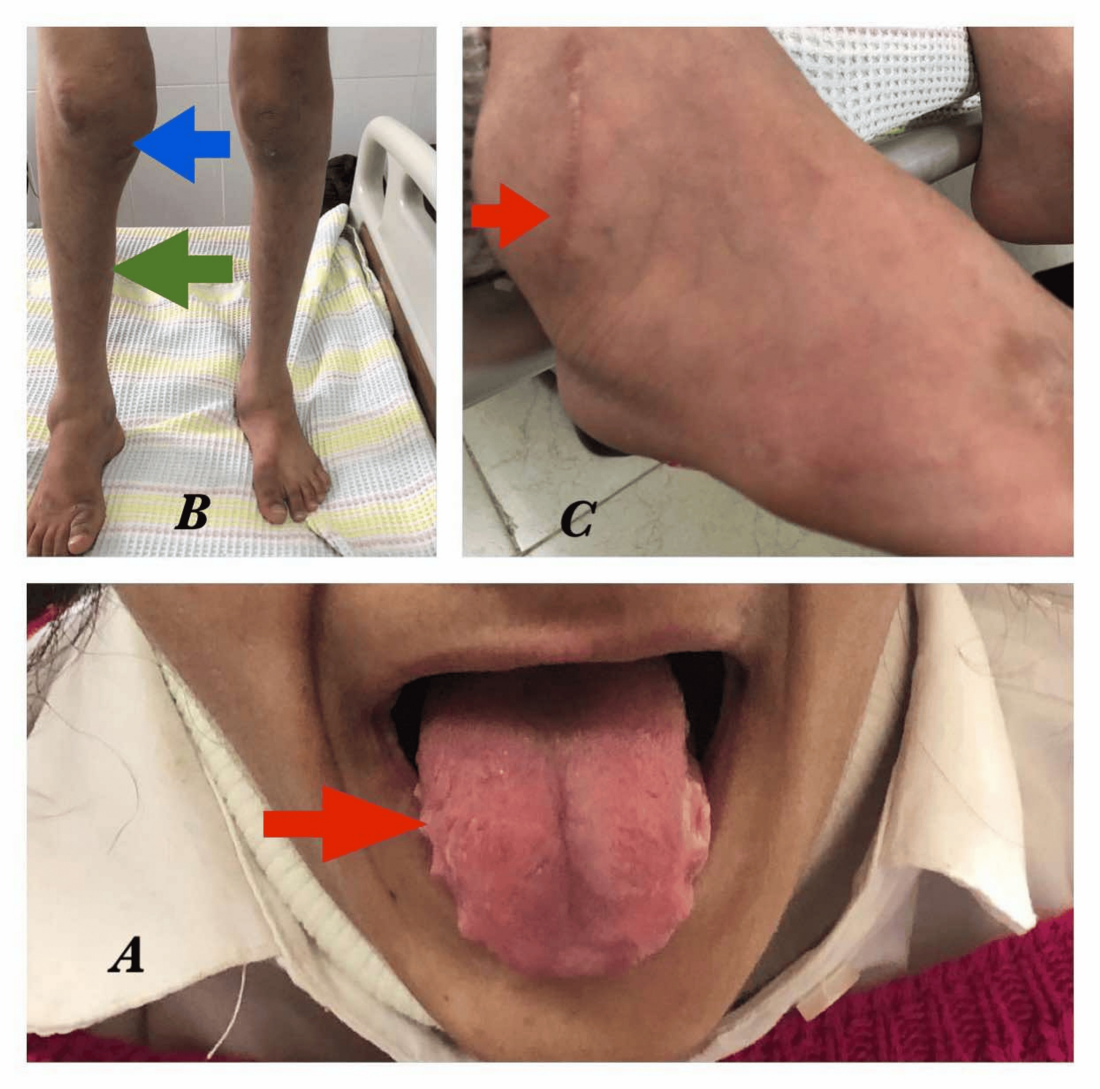
(A) Mouth ulcers and tongue biting (red arrow). (B) Right knee swelling (blue arrow) and muscle atrophy (green arrow) were present. (C) Charcot joint (the Charcot joint describes a degenerative/destructive joint disease that progresses over time in individuals who have impaired proprioception and pain perception) and scar on the right ankle (red arrow).

At the age of four, the patient's parents noticed that she was falling from various heights without experiencing any pain, and her father claimed that this bothered them. She displayed signs of pain insensitivity such as unsightly sores on her lower limbs, mouth ulcers, and tongue biting (Figure 1).

The patient complained of right knee swelling accompanied by limping throughout the course of 4 to 10 years, as well as many stages of wound healing at various places (Figure 2). The patient also displayed muscular atrophy, which was more pronounced at the lower extremities. Anosmia, epilepsy, or febrile seizures were unreported. Upon examination, the child was found to have multiple scars all over the body, severe muscle atrophy predominantly in lower limbs, and the Charcot joint, described as a degenerative/destructive joint disease that progresses over time in individuals who have impaired proprioception and pain perception (Figure 3). According to this presentation, investigations included CBC, rheumatological markers (rheumatoid factor, antinuclear antibody, anti-cyclic citrullinated peptide antibody, etc.), Brucella, and EMG, without any significant results. A right ankle X-ray showed an osteolytic lesion in the lateral side of the right femur epiphysis. Following that, the right knee MRI revealed bony erosion at the lateral femoral epicondyle with multiple bony fragments, subchondral cystic changes, and mild joint effusion. MRI of the ankle revealed bony erosion, bony fragments, and joint effusion. Bone scan revealing chronic inflammatory changes in the right knee and ankle. Synovial fluid analysis revealed the following: turbid, red blood cell count 60,000, white blood cell count 1,300, lactate dehydrogenase (LDH)/fluid 219, LDH/serum 208, LDH ratio 1.05, protein/fluid 4.2, protein/serum 7.9, and protein ratio 0.53. A tissue biopsy from the right knee revealed synovium infiltrated with inflammatory cells. The genetic testing for CIPA - PRDM 12 and NTKR1 - was both negative. However, the patient was confirmed as having CIPA since the whole exome sequences (WES) identified a homozygous *SCN9A* probable pathogenic variation c.901A>T p.(Lys301*), which results in a premature stop codon. Although parents were second cousins, there was no family history for CIPA.

## Discussion

CIPA is a nonsense mutation that has the potential to have significant consequences; therefore, early identification of the etiology and assistance with symptom relief is essential for effective care and preventative measures [3].

All sensory neurons express the *SCN9A* gene, which is a crucial component in the processing of peripheral pain. This gene encodes a voltage-gated sodium channel (Nav 1.7), which is important in nociceptive signaling and has both gain and loss-of-function mutations. It is interesting to note that the phenotype that results from a given mutation might vary significantly [4]. Thus, loss-of-function mutations in the *SCN9A *gene result in the truncation of the sodium channel Nav 1.7 protein, which causes channelopathy-associated autosomal recessive congenital pain sensitivity [5].

Given the anticipated effects of the alteration in the *SCN9A* gene in our patient, a disease causing a new variant of *SCN9A* with a presentation not previously described in the literature is likely to be the cause. The literature states that the NTRK1 [6] variant typically manifests with anhidrosis, the propensity to develop corneal ulcers that heal poorly [7], intellectual disability in the majority of patients, predisposition to staph aureus infections, Charcot joints, and dry skin with or without lichenification [8]. The *SCN9A* variant typically manifests with anosmia, Charcot joints, and normal corneal reflex and tear production [8-12].

As a result, our patient with the *SCN9A* mutation displayed characteristics of CIPA rather than anosmia, Additionally, there are abnormalities of the ankles, anhidrosis, arthritis, low body weight, stunted growth, fever, hyperpigmentation of the skin, leukopenia, pain intolerance, scarring, self-mutilation, and thickened skin.

Knowing the key characteristics to take into account in the differential diagnosis in pediatric practice is crucial given their uncommon occurrences and the few cases that have been documented [13]. Uncertainty surrounds the CIPA's therapeutic strategy [14]. The only course of action for treating CIPA is still prevention of complications, morbidities, and the disease (genetic counselling in pregnancy perhaps) [14]. Early surgical treatment for long bone fractures reduces the risk of future osteopenia by allowing early weight-bearing and preventing pseudo-arthrosis [15]. The selection of the best management in situations of infection may stop additional joint damage [15].

## Conclusions

CIP syndrome (SCN9A) is a rare clinical event, making research on it scarce. However, because of its serious complications, therapeutic interventions and recommendations generally include early diagnosis and interventions, regular follow-up, social support for patients and families, consistent physiotherapy, patient/caregiver education, expanding knowledge of genetic and molecular models of pain, which must be taken into consideration in the differential diagnosis of recurrent arthritis and recurrent episodes of unexplained fever. These patients require comprehensive care to enhance their quality of life and avoid suffering unintended harm or passing away.
